# Iptacopan combined with immunosuppressive therapy for aplastic anemia-paroxysmal nocturnal hemoglobinuria syndrome: A case report

**DOI:** 10.1097/MD.0000000000051162

**Published:** 2026-10-16

**Authors:** Xiaoqian Liu, Jie Xu, Yaqin Luo, Yingying Wang, Jianping Li, Linying Li, Zhaoxia Liu

**Affiliations:** a The First Clinical Medical College, Shandong University of Traditional Chinese Medicine, Jinan, Shandong, China; b Department of Hematology, Affiliated Hospital of Shandong University of Traditional Chinese Medicine, Jinan, Shandong, China.

**Keywords:** aplastic anemia-paroxysmal nocturnal hemoglobinuria syndrome, case report, immunosuppressive therapy, iptacopan

## Abstract

**Rationale:**

Aplastic anemia-paroxysmal nocturnal hemoglobinuria (AA-PNH) syndrome is a rare acquired hematopoietic stem cell disorder. Patients who respond poorly to conventional immunosuppressive therapy (IST) and corticosteroids face limited therapeutic options and a poor prognosis. This article reports on the use of iptacopan combined with IST for AA-PNH syndrome.

**Patient concerns:**

A previously healthy 24-year-old Han Chinese male presented with dizziness and fatigue and was found to have pancytopenia. He was initially treated with cyclosporine. However, 2 years after discontinuation, he developed acute hemolysis. After treatment with iptacopan, the patient’s hemolysis was quickly controlled, but he still required blood transfusions.

**Diagnoses:**

The patient was initially diagnosed with aplastic anemia through bone marrow examination, along with a paroxysmal nocturnal hemoglobinuria clone. After recurrent acute hemolysis, reexamination of the bone marrow and flow cytometry revealed partial deficiency of cluster of differentiation (CD) 55/CD59, and fluorescently labeled aerolysin-negative cells accounted for more than 90% of granulocytes and monocytes. Eventually, the diagnosis of AA-PNH syndrome was made.

**Interventions:**

After acute hemolysis was initially managed with steroids and IST, the patient was started on iptacopan to achieve rapid hemolysis control. Given persistent transfusion dependence due to bone marrow hypoplasia, immunosuppressants and hematopoietic stimulants (danazol and eltrombopag) were added while continuing iptacopan. The hematopoietic stimulants were discontinued after 3 months due to abnormal liver function tests, and the patient was maintained on iptacopan combined with IST.

**Outcomes:**

Iptacopan rapidly and effectively controlled hemolysis, and no breakthrough hemolysis, extravascular hemolysis, or drug-related adverse events were observed. Critically, iptacopan served as a bridge to control hemolysis, while the subsequent addition of IST (tacrolimus/cyclosporine) addressed the underlying autoimmune bone marrow failure. After combined treatment with iptacopan and IST, the patient’s blood cell count gradually recovered, hemoglobin returned to normal levels, and blood transfusion was no longer required.

**Lessons:**

This case suggests that iptacopan combined with IST may represent a potentially effective and safe therapeutic option for AA-PNH syndrome that is refractory to conventional IST and corticosteroids. Especially for AA-PNH patients with impaired bone marrow function and continuous dependence on blood transfusion, iptacopan combined with IST may be an effective and well-tolerated treatment regimen.

## 1. Introduction

Aplastic anemia-paroxysmal nocturnal hemoglobinuria (AA-PNH) syndrome refers to a clinical syndrome in which patients simultaneously or sequentially exhibit both aplastic anemia (AA) and paroxysmal nocturnal hemoglobinuria (PNH). These patients may present with clinical and laboratory features of both bone marrow failure and complement-mediated intravascular hemolysis. Studies have found that more than half of AA patients tested positive for PNH clones via flow cytometry at initial diagnosis. Most AA-PNH patients show significant improvement after treatment with immunosuppressive therapy (IST) combined with hematopoietic stimulants. However, for patients with a high proportion of PNH clones or those who respond poorly to IST alone and exhibit significant hemolysis, the combined use of IST and complement inhibitors can markedly enhance therapeutic efficacy. Here, we report a case of AA-PNH syndrome effectively treated with iptacopan combined with IST. The patient had no prior history of complement inhibitor therapy. After treatment with iptacopan and IST, hemolysis was effectively controlled, anemia significantly improved compared with pretreatment levels, hemoglobin (Hb) returned to normal, and the patient achieved platelet (PLT) transfusion independence.

## 2. Case report

A 24-year-old male with no significant past medical history, a healthy lifestyle, good mental health, and stable social relationships reported no history of exposure to radiation or toxic substances. There was no notable family history of genetic disorders. In April 2018, he presented with dizziness and fatigue and was found to have pancytopenia. A subsequent bone marrow examination led to a diagnosis of AA with a PNH clone. He was treated with oral cyclosporine for 1 year, which was tapered and discontinued after his hematologic parameters normalized. In September 2021, he experienced an acute hemolytic episode and was managed with oral glucocorticoids, which maintained his Hb at approximately 90 g/L and PLT count around 60 × 10^9^/L.

In November 2021, the patient presented with fever and dark brown (cola-colored) urine. Physical examination revealed no abnormal pulmonary signs or peripheral lymphadenopathy. Laboratory investigations were significant for the following: Hb 53 g/L, PLT 45 × 10^9^/L, reticulocyte percentage (Ret) 4.2%, fibrinogen 2.86 g/L, D-dimer 2.34 μg/mL, aspartate aminotransferase (AST) 91 U/L, total bilirubin (TBIL) 50.8 μmol/L, indirect bilirubin (IBIL) 44.2 μmol/L, and lactate dehydrogenase (LDH) 3319 U/L. A diagnosis of acute hemolysis complicated by an upper respiratory tract infection and hepatic impairment was made. The hemolytic crisis was controlled following treatment with sodium bicarbonate, glucocorticoids, hepatoprotective agents, and antimicrobial therapy.

A bone marrow evaluation was performed on December 9, 2021. Morphological examination showed active granulocytic and erythroid hyperplasia with normal proportions and morphology. Only 2 megakaryocytes were observed in the entire smear, and PLTs were scarce. Bone marrow biopsy indicated mildly hypocellular marrow with a 35% hematopoietic area. Granulocytic and erythroid series showed mature differentiation, with a slightly reduced number of megakaryocytes. No significant dysplasia or increase in blasts was observed. Reticulin fiber staining was negative, and iron staining was positive. Flow cytometry analysis for cluster of differentiation (CD) 55 and CD59 revealed normal expression of CD55 (99.9%) and partial loss of CD59 (43.4%) on red blood cells. Partial loss of both CD55 (14.1%) and CD59 (14.1%) was detected on granulocytes. Fluorescently labeled aerolysin (FLAER) testing showed that FLAER-negative cells accounted for 91.54% of monocytes and 90.16% of granulocytes. Immunophenotyping by flow cytometry demonstrated abnormal phenotypes in monocytes and granulocytes, with an increased proportion of nucleated red blood cells. A diagnosis of AA-PNH syndrome was confirmed. After 1 month of corticosteroid therapy, follow-up tests showed Hb 80 g/L, PLT 36 × 10^9^/L, LDH decreased to 1433 U/L, and IBIL decreased to 39.2 µmol/L. An unrelated human leukocyte antigen-matched donor search in the China Marrow Donor Program was unsuccessful. The patient was discharged on oral tacrolimus combined with corticosteroids and remained stable.

In July 2024, the patient again presented with yellowish complexion, fatigue, and tea-colored urine, indicating an acute hemolytic episode. Laboratory tests revealed the following: white blood cell count (WBC) 1.39 × 10^9^/L, absolute neutrophil count (ANC) 0.95 × 10^9^/L, Hb 58 g/L, PLT 17 × 10^9^/L, Ret 6.6%, fibrinogen 1.86 g/L, D-dimer 1.19 µg/mL, AST 59 U/L, TBIL 83 µmol/L, IBIL 69.3 µmol/L, glucose 17.24 mmol/L, and LDH 1370 U/L. High-sensitivity PNH testing detected PNH clones in 46.35% of red blood cells, 87.91% of granulocytes, and 89.50% of monocytes. On July 17, 2024, the patient was initiated on oral iptacopan hydrochloride capsules (200 mg, twice daily). After 1 week of treatment, Hb increased to 68 g/L, LDH decreased to 410 U/L, IBIL was 40.8 µmol/L, Ret decreased to 4%, and serum glucose returned to normal levels. At 4 weeks of treatment, Hb further improved to 86 g/L, LDH decreased to 268 U/L, and IBIL was 19.1 µmol/L. These findings indicate that the acute hemolysis was rapidly controlled, with a marked increase in Hb and a decrease in reticulocyte count, LDH, and IBIL to near-normal levels.

On August 26, 2024, the patient developed symptoms of respiratory tract infection, including rhinorrhea, sore throat, and mild cough, following exposure to cold. No tea-colored or cola-colored urine was observed. The symptoms improved rapidly after oral antibiotic treatment. However, follow-up complete blood count revealed the following: WBC 0.79 × 10^9^/L, ANC 0.23 × 10^9^/L, Hb 68 g/L, Ret 2.5%, PLT 11 × 10^9^/L, LDH 273 U/L, and IBIL 19.4 µmol/L. The Coombs test was negative. Treatment was adjusted with the addition of danazol (0.6 g/d, orally in 3 divided doses) and recombinant human granulocyte colony-stimulating factor (150 µg/d, subcutaneously). The neutrophil count showed a therapeutic response, but Hb continued to decline progressively. A repeat bone marrow evaluation was performed on September 13, 2024. Bone marrow morphology showed markedly hypercellular marrow with active granulopoiesis and normal morphology; active erythropoiesis with enlarged central pallor in some erythrocytes, and frequent macrocytes and polychromatic erythrocytes; lymphocytes accounted for 20% without significant morphological abnormalities; and 5 megakaryocytes were observed in the entire smear. Bone marrow biopsy revealed relatively hypocellular marrow (approximately 20% hematopoietic tissue), adipocyte hyperplasia, and small focal aggregates of granulocytic and erythroid cells; no megakaryocytes were identified. Special stains: reticulin fiber (MF-0) and PAS (+). Immunohistochemistry: CD34+ (<1%), CD117+ (approximately 1%), and CD163+. Leukemia immunophenotyping: myeloblasts (CD34+ CD117+) accounted for 0.05%; loss of CD14 antigen expression on monocytes. High-sensitivity PNH testing detected PNH clones in 46.32% of red blood cells, 85.61% of granulocytes, and 80.02% of monocytes. Testing for myelodysplastic syndrome-related gene mutations was negative, and chromosomal karyotype analysis revealed a normal karyotype. Diagnosis: AA-PNH syndrome. Treatment was continued with oral iptacopan and danazol, with the addition of the immunosuppressant tacrolimus (2 mg orally twice daily) and the thrombopoietin receptor agonist eltrombopag (75 mg orally once daily). Transfusions of red blood cells and PLTs were administered to alleviate anemia and bleeding symptoms. On December 21, 2024, danazol and eltrombopag were discontinued due to abnormal liver function, while oral iptacopan and tacrolimus were continued. Hepatoprotective therapy was initiated concurrently.

On February 10, 2025, follow-up complete blood count revealed the following: WBC 1.86 × 10^9^/L, ANC 1.10 × 10^9^/L, Hb 92 g/L, PLT 22 × 10^9^/L, AST 23 U/L, TBIL 45.8 µmol/L, IBIL 15.1 µmol/L, and LDH 311 U/L. The patient had not required red blood cell or PLT transfusions for 2 months. Subsequently, per the patient’s request, tacrolimus was discontinued and replaced with cyclosporine (100 mg orally twice daily). As of May 11, 2025, the patient’s laboratory results were as follows: WBC 3.04 × 10^9^/L, ANC 1.85 × 10^9^/L, Hb 128 g/L, PLT 27 × 10^9^/L, Ret 2.67%, AST 21 U/L, IBIL 29.1 µmol/L, and LDH 266 U/L. Changes in Hb and PLT before and after treatment with iptacopan, as well as before and after the addition of tacrolimus, are shown in Figure 1; corresponding changes in LDH and IBIL levels are presented in Figure 2. The overall timeline of the patient’s treatment course is summarized in Figure 3.

**Figure 1.**
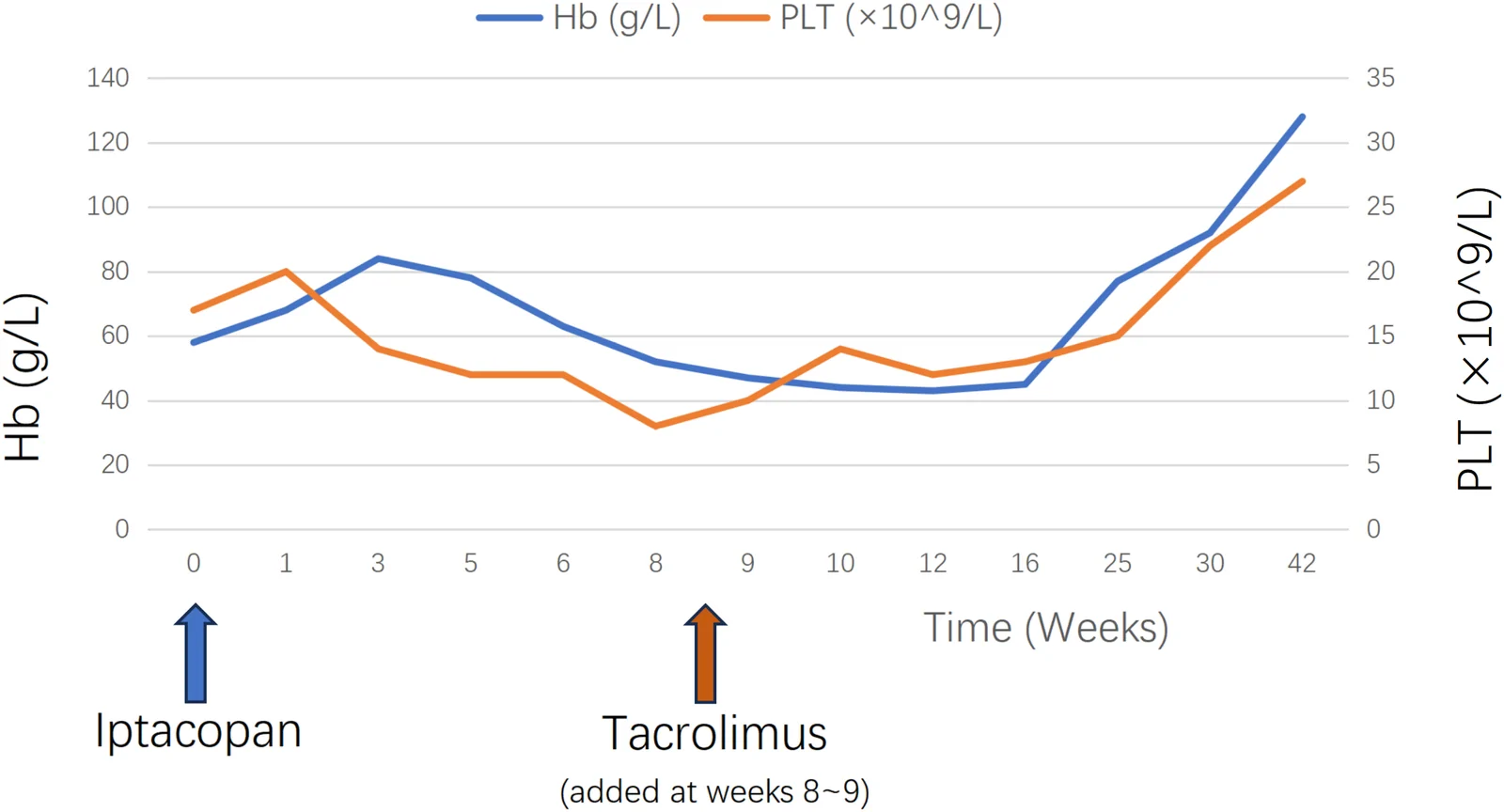
Changes in hemoglobin (Hb) and platelet (PLT) count before and after iptacopan treatment, and before and after the addition of tacrolimus.

**Figure 2.**
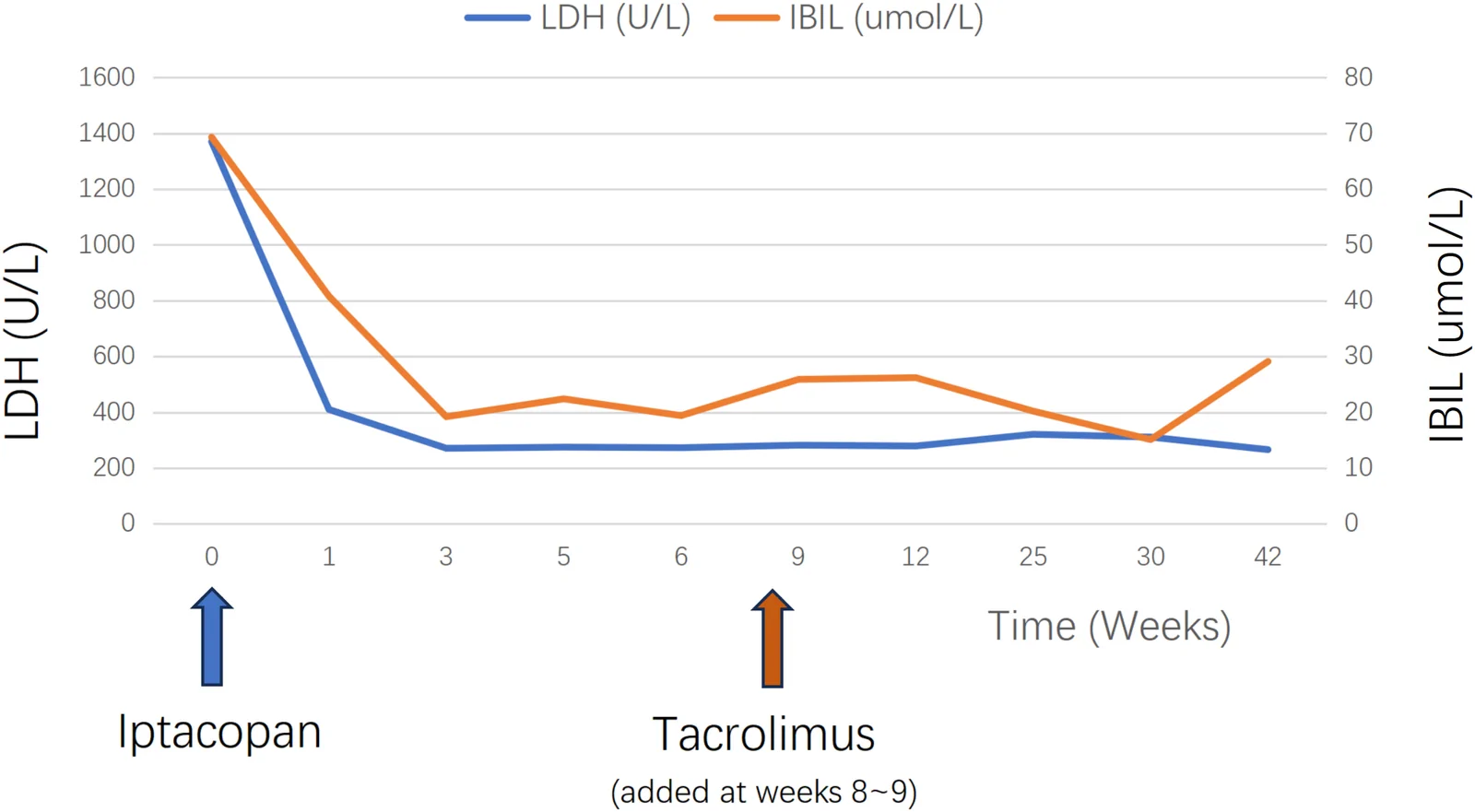
Changes in lactate dehydrogenase (LDH) and indirect bilirubin (IBIL) levels before and after iptacopan treatment, and before and after the addition of tacrolimus.

At the final follow-up in August 2025, after 12 months of iptacopan therapy, the patient reported adherence to the prescribed regimen and a significant improvement in physical strength. Throughout the treatment course, no episodes of breakthrough or extravascular hemolysis occurred. Furthermore, no treatment-related adverse events, such as dizziness, nausea, anorexia, abdominal distension, or fever, were observed.

## 3. Discussion

Both PNH and AA are benign hematologic disorders that can transform into each other under various influences. Studies have shown that the pathogenesis of PNH and AA is associated with T-cell-mediated autoimmune processes, indicating a shared immunopathological basis.^[1]^ The term AA-PNH syndrome is applied when one disease transforms into the other, or when features of both are present simultaneously. Up to 70% of AA patients have a PNH clone at initial diagnosis, and 40% of PNH cases evolve from AA.^[2]^ Modern medicine suggests that the mechanism of AA progressing to secondary PNH primarily involves immune escape, clonal selection, and disease progression. Initially, in AA patients with a PNH clone, abnormally activated T cells target hematopoietic stem cells (HSCs) by recognizing glycosylphosphatidylinositol-anchored proteins. However, HSCs carrying the PNH clone lack glycosylphosphatidylinositol-anchored proteins due to phosphatidylinositol glycan anchor biosynthesis class A gene mutations. As a result, normal HSCs in AA patients are attacked by T cells, while PNH clone-bearing HSCs escape immune attack and gain a survival advantage, leading to clonal expansion under immune pressure in AA.^[1]^ In this case, the loss of CD55/CD59 expression on granulocytes (14.1% → 87.9%) demonstrates a typical process of clonal expansion. On the other hand, the use of immunosuppressants is also an important factor promoting the proliferation of PNH clone cells. Several studies have shown that PNH clones significantly increase in AA patients after IST. Moreover, it is relatively uncommon for patients without detectable clones before treatment to develop PNH clones after IST, further supporting the association between AA progressing to PNH and immune escape of PNH-positive cells.^[3]^ Additionally, the inflammatory microenvironment in AA, long-term immune damage to HSCs, and impaired repair mechanisms may promote the accumulation of phosphatidylinositol glycan anchor biosynthesis class A gene mutations. Ultimately, under immune pressure driven by multiple factors, defective PNH clone cells are selectively expanded through competition, gradually replacing normal hematopoiesis, leading to complement-mediated hemolysis and the development of AA-PNH syndrome.

There is currently no unified consensus on the treatment of AA-PNH syndrome. IST may be used for patients with PNH accompanied by bone marrow failure; however, conventional IST cannot inhibit complement-mediated hemolysis and may carry the risk of promoting PNH clone expansion. Allogeneic HSC transplantation remains the only curative treatment for AA or PNH. Nevertheless, in the era of complement inhibitors, the indications for HSC transplantation are limited to PNH patients with severe refractory bone marrow failure, potential progression to high-risk myeloid neoplasms, or inadequate response to complement inhibitor therapy. Additionally, its application is often constrained by challenges such as donor availability and thrombotic complications.

Complement inhibitors, by targeting the abnormal activation of the complement system, can rapidly control acute hemolytic episodes and prevent PNH-related complications. They have become essential agents for treating AA-PNH patients presenting with acute intravascular hemolysis and organ dysfunction. Eculizumab, a monoclonal antibody against terminal complement component C5, was the first complement inhibitor approved for PNH. It binds to C5, preventing its cleavage into C5a and C5b, thereby reducing the formation of the membrane attack complex and controlling intravascular hemolysis. However, although terminal complement inhibition at the C5 level effectively controls intravascular hemolysis and reduces thrombosis in PNH patients, it does not adequately suppress extravascular hemolysis. This is because while C5 inhibitors prevent C5 cleavage, early steps of complement activation may still occur. The C3 cleavage fragment C3b continues to be generated and accumulates on GPI-deficient red blood cells, leading to opsonization and subsequent phagocytosis of C3b-coated PNH red blood cells by macrophages in the liver and spleen, resulting in extravascular hemolysis. Studies have shown that almost all PNH patients treated with eculizumab experience C3b-mediated extravascular hemolysis, leading to persistent anemia and transfusion dependence in a significant proportion of patients.^[4]^ Additionally, challenges such as intravenous administration, frequent dosing, and disease relapse after discontinuation pose significant burdens to treatment.

In contrast to commonly used C5 complement inhibitors like eculizumab, iptacopan targets the proximal pathway of the alternative complement system by specifically inhibiting complement factor B, serving as an oral-specific inhibitor of factor B. In the alternative pathway, factor B is a key protein for complement activation. Under the action of factor D, factor B is cleaved into fragment Bb, which binds to C3b to form the C3 convertase, further activating downstream complement cascades. Moreover, the feedback amplification loop mediated by factor B in the alternative pathway rapidly amplifies complement signals. By specifically inhibiting factor B, iptacopan prevents the formation of C3 convertase, inhibits membrane attack complex generation, and thereby reduces intravascular hemolysis. Due to impaired C3 convertase formation, it also decreases deposition of C3b on cell surfaces, reducing macrophage-mediated phagocytosis and lysis of red blood cells, thus mitigating extravascular hemolysis. Therefore, iptacopan provides comprehensive control of both intravascular and extravascular hemolysis by blocking proximal complement activation at the factor B level, which prevents C3b opsonization and subsequent phagocytosis of PNH red blood cells. Furthermore, compared with intravenous complement inhibitors, oral administration of iptacopan offers greater convenience. For PNH patients who are complement inhibitor-naïve or have a suboptimal response to anti-C5 therapy, iptacopan monotherapy may become a preferred treatment option in the future.^[5]^

The present case is a typical AA-PNH syndrome. After multiple hemolytic episodes, the patient was treated with iptacopan. Within 1 week, Hb increased by 10 g/L and LDH decreased by 69%. By week 4, Hb reached 86 g/L and LDH approached normal levels, demonstrating a faster onset of action compared with eculizumab.^[6]^ Meanwhile, the PNH clone size slightly decreased after treatment, consistent with the potential effect of iptacopan in inhibiting clonal proliferation. Follow-up high-sensitivity PNH testing at 12 months was not performed; therefore, the true PNH clone size under iptacopan maintenance therapy could not be assessed. However, it is important to distinguish the distinct roles of each therapeutic component in this case. Iptacopan rapidly and effectively controlled intravascular hemolysis (as reflected by the sharp decline in LDH and IBIL), but it did not immediately reverse the underlying bone marrow failure. The patient remained transfusion-dependent for red cells and PLTs despite excellent hemolysis control. It was only after the addition of tacrolimus in September 2024 that trilineage hematopoietic recovery was observed, with Hb gradually rising to 128 g/L by May 2025. This temporal sequence strongly suggests that while iptacopan served as a critical bridge to control hemolysis, the recovery of endogenous hematopoiesis was primarily driven by IST targeting the autoimmune stem cell injury. No adverse reactions were observed when iptacopan was combined with tacrolimus/cyclosporine in this case, and Hb eventually recovered to 128 g/L, indicating its dual benefits in managing bone marrow failure and hemolysis. Furthermore, the patient received a 3-month course of eltrombopag (September–December 2024) concurrently with tacrolimus. Given that eltrombopag has a well-recognized delayed effect on HSC expansion, often requiring 3 to 6 months to show trilineage improvement, it is clinically plausible that the hematopoietic response observed in early 2025 was partially primed by this course of eltrombopag. This confounding factor should be considered when interpreting the contribution of IST alone to hematologic recovery. However, it should be noted that the PNH clone in this case was predominantly in granulocytes/monocytes, which may influence differences in treatment response.

Synchronous treatment of PNH and AA is relatively uncommon in clinical practice. Current literature and research predominantly focus on eculizumab combined with IST. A UK retrospective study evaluated the efficacy and safety of eculizumab administered either concurrently with IST or within 1 year of initiating AA treatment in 25 PNH-positive patients with bone marrow failure. The results demonstrated that the combination therapy did not adversely affect treatment efficacy or safety.^[7]^ Two additional international and European observational studies found that regardless of the sequence of administration, eculizumab combined with IST had no negative impact on treatment outcomes and raised no safety concerns.^[8,9]^ Expert consensus recommends that for PNH patients with underlying bone marrow failure disorders such as AA, the therapeutic focus should generally remain on the primary bone marrow disease. The presence of a PNH clone should not directly influence the treatment strategy for bone marrow failure. However, when AA patients develop PNH clonal expansion accompanied by hemolysis or thrombosis, they should be reclassified as having classic PNH and receive complement inhibitor therapy.^[10]^

Iptacopan offers a new therapeutic option for patients with AA-PNH syndrome. Compared with the widely used eculizumab, iptacopan provides the advantage of simultaneously controlling both intravascular and extravascular hemolysis. Its combination with IST may emerge as a primary treatment strategy for AA-PNH syndrome in the future. However, the long-term safety of combining iptacopan with IST, as well as its efficacy across different PNH clone subtypes, still requires validation through multicenter, large-sample clinical trials. Future research should focus on the long-term inhibitory effects of iptacopan on PNH clones, the optimal timing for combination with IST, and its potential application in patients with severe bone marrow failure. In particular, prospective studies with standardized follow-up flow cytometry are needed to evaluate the true impact of iptacopan on PNH clone size over time. These efforts could advance the treatment paradigm for AA-PNH syndrome from symptomatic control toward synergistic and potentially curative approaches.

**Figure 3.**
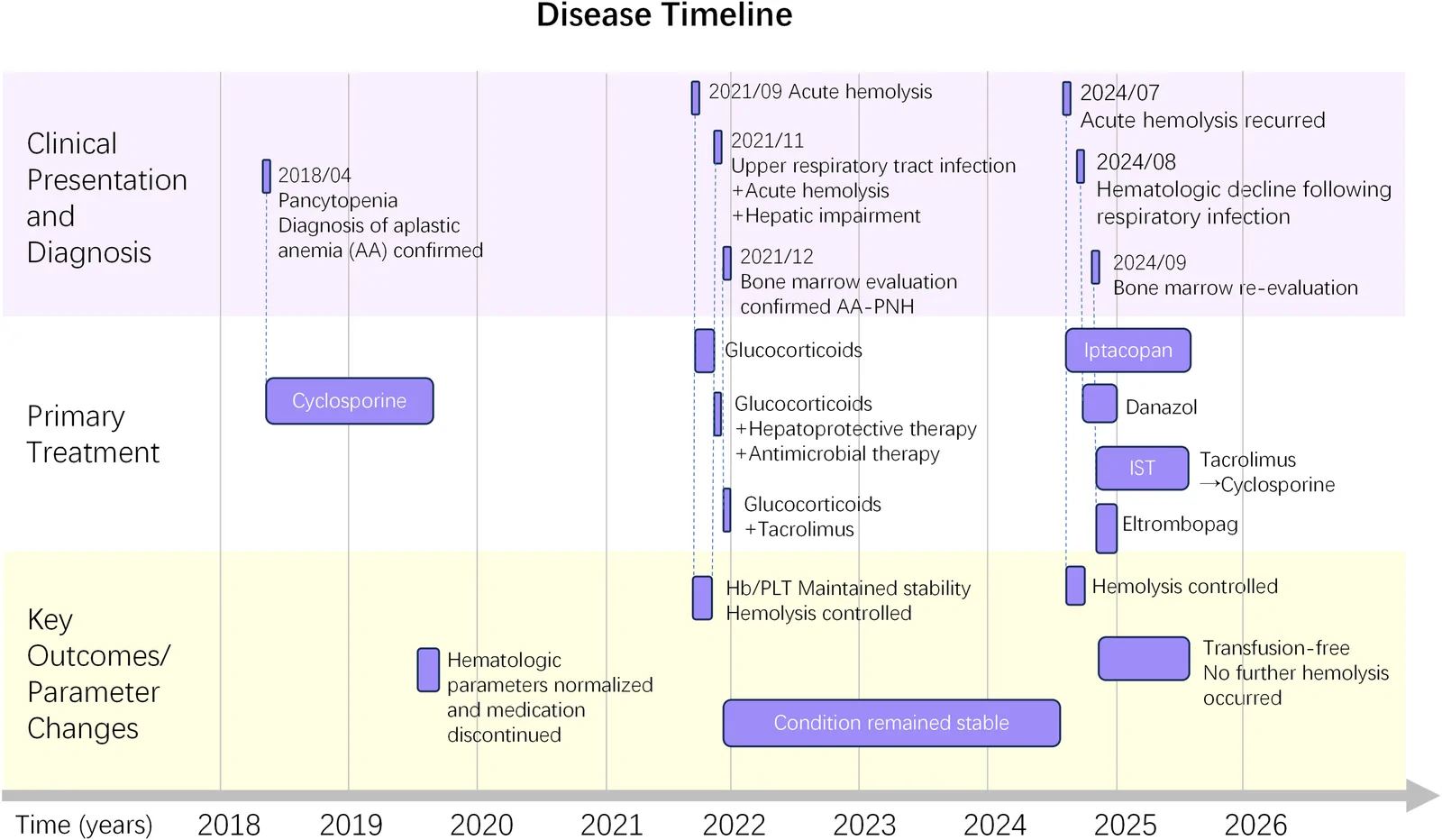
Historical and current information from this episode of care (timeline of diagnosis, treatments, and key clinical events). AA-PNH = aplastic anemia-paroxysmal nocturnal hemoglobinuria, Hb = hemoglobin, PLT = platelet.

## Author contributions

**Conceptualization:** Xiaoqian Liu, Jie Xu, Yaqin Luo, Zhaoxia Liu.

**Data curation:** Xiaoqian Liu, Yingying Wang, Jianping Li.

**Investigation:** Xiaoqian Liu, Linying Li.

**Writing – original draft:** Xiaoqian Liu.

**Writing – review & editing:** Zhaoxia Liu.
